# Consistency–accuracy correlation in hard-prompted LLMs for entity and relation extraction: empirical findings from plant-health data

**DOI:** 10.1186/s44342-025-00063-2

**Published:** 2026-02-10

**Authors:** Xinzhi Yao, Claire Nédellec, Jingbo Xia, Robert Bossy

**Affiliations:** 1 National Research Institute for Agriculture, Food and Environment, University Paris-Saclay, MaIAGE, Jouy-en-Josas, France; 2 College of Informatics, Hubei Key Laboratory of Agricultural Bioinformatics, Huazhong Agricultural University, Wuhan, China

**Keywords:** Relational information extraction, Large Language Model evaluation, Prompting, Consistency, Accuracy, Plant health, Document-level information extraction

## Abstract

As large language models (LLMs) become increasingly popular for information extraction (IE), concerns persist regarding the stability and reliability of their outputs. While accuracy has traditionally been the main evaluation metric, consistency—defined as the stability of model outputs across repeated runs—has recently been proposed as a complementary signal of reliability. In this work, we examine the relationship between accuracy and consistency in hard-prompted generative LLMs applied to entity and relation extraction. We conduct a systematic evaluation using four LLMs (GPT, DeepSeek, Qwen, Kimi) on the EPOP corpus, a plant-health dataset with rich entity types, long-range relations, overlapping relations, and strong argument constraints. To refine the interpretation of consistency, we distinguish between recoverable output variations—those that preserve the meaning of the extracted information—and critical ones that result in semantic errors. Our results show that while some positive correlation between accuracy and consistency exists, it is model-dependent and varies with task complexity. In structured prediction tasks, we show that consistency should be measured at the semantic level, ignoring superficial variations in format or wording. These insights have important implications for using self-consistency as a confidence filter and for designing reliable generative IE pipelines in specialized domains.

**Supplementary Information:**

The online version contains supplementary material available at 10.1186/s44342-025-00063-2.

## Introduction

Information extraction (IE) is a core task in natural language processing (NLP), enabling the automatic identification and structuring of informational content—such as entities, relations, and events—from unstructured text. Its applications are central to knowledge base construction, question answering, scientific discovery, and downstream reasoning tasks. With the increasing scale and complexity of textual data, especially in domains such as biomedical and environmental sciences, accurate IE has become more crucial than ever. In recent years, generative large language models (LLMs) have been widely adopted across NLP tasks including relational information extraction. Their flexibility and zero- or few-shot capabilities make them appealing alternatives to task-specific architectures and low-resources. Unlike traditional approaches that rely on classification or sequence labeling pipelines, generative models produce outputs as free-form text, opening new possibilities but also introducing new challenges.

One key challenge is the inherent unpredictability of generative models, which can produce inconsistent outputs even when given the same input. This stochastic behavior undermines the reliability of LLMs, especially in applications where reproducibility and factual accuracy are critical. Traditionally, model quality has been evaluated by accuracy on representative test sets. Yet, as generative models are increasingly deployed in open-ended and real-world scenarios, this raises an important question: *is accuracy on representative data still a sufficient indicator of a model’s performance at inference time?*

To address this, recent research has explored consistency—defined as the stability of model outputs under repeated sampling—as a potential indicator of reliability [1]. Notably, self-consistency techniques generate multiple outputs and select the most frequent one, under the assumption that consistency correlates with correctness [2]. However, this assumption is not guaranteed: a model can be confidently and consistently wrong [3].

This paper investigates two research questions in this context: is consistency positively correlated with accuracy in relational IE tasks? If so, consistency could serve as a lightweight and model-agnostic filter for reliable predictions at inference time. If not, the usefulness of consistency as a filtering method must be reconsidered.

What do accuracy and consistency mean in the context of generative IE? In contrast to classification tasks with fixed outputs, relational IE may have multiple correct extractions, especially when phrased differently, but semantically equivalent [4]. This variability can reduce consistency and accuracy measured by classical metrics while still fulfilling the information need. This raises important questions about metric design. For example, constraints that are trivial for non-generative systems (e.g., output format or type correctness) may be harder to enforce by generative models. This prompts a deeper investigation: which evaluation criteria should be preserved, and which should be relaxed or redefined for generative settings? To answer, we distinguish variation types in the generated outputs by analyzing whether they affect the final result, i.e., whether the variation is recoverable (preserving the semantic content) or disruptive.

Beyond this central question, we also explore the factors that contribute to output variability in generative models for relational IE. Specifically, we examine whether consistency is influenced by the length of the input or output or the inherent complexity of the task [5]. By systematically analyzing these variables, we aim to better understand when and how consistency can be a meaningful signal for prediction quality in generative relation extraction.

To explore these questions, we conduct an in-depth analysis of the relationship between consistency and accuracy in generative models applied to a zero-shot document-level relation extraction (DocRE) challenging task [6]. DocRE was preferred over text-bound information extraction tasks in order to limit the task to a single-step generation process. We use hard prompting to minimize training influence and isolate inference-time variability. We experiment with four popular LLMs—Kimi, DeepSeek, GPT, and Qwen—on the Epidemiomonitoring of Plant (EPOP) corpus, which was selected because it reflects broad goals of relational information extraction. This corpus presents a challenging and realistic setting that includes lexical named entities and their normalization into semantic classes (to mitigate NER variability), long-distance relationships, and structured schema with strict argument type constraints.

This experimental setup enables us to assess how different models handle linguistic and structural variation, and how such variation impacts accuracy and consistency. Ultimately, our work aims to clarify the role of consistency in evaluating relational generative IE systems and inform the design of future evaluation protocols.

The consistency of generation in large language models (LLMs) has emerged as a key indicator of overall model reliability. Atil et al. (2024) [7] showed that even under deterministic settings (e.g., temperature parameter set to zero), repeated inferences with the same input can yield divergent outputs across models. To quantify this variability, they introduced two metrics: the Token Agreement Ratio on raw output (TARr), which measures agreement across the entire generated strings, and TARa, which focuses on the answer part of the output. The latter TARa requires post-processing to extract the relevant answer segment from the full output. Lee et al. (2024) [8] examined LLMs as evaluators, introducing *self-consistency* (under fixed rating scales) and *cross-scale consistency* (across varying scales), and found that many commercial models produce inconsistent ratings in the same task under different conditions [9] proposed the *internal consistency* concept, emphasizing stable expression of knowledge across response, decoding, and latent levels. The authors argued that “Consistency Is (Almost) Correctness”: while consistency can amplify errors, it more often reinforces correct outputs due to the predominance of accurate data in training corpora. Despite these advances, consistency evaluation in relational or structured information extraction (IE) tasks remains underexplored. Although TARa assesses answer-level consistency, it overlooks the structural constraints specific to relational information extraction, such as constraints on entity and relation types and boundary alignment.

## Methods

### Task

We conducted document-level relation extraction experiments using hard prompting as this task highlights the strengths of LLM-based Information Extraction, particularly their ability to perform deep and broad document understanding without requiring fine-tuning. Conversely, LLMs have shown limitations when prompted to pinpoint the exact grounding of text of named entities and relations, or to normalize entities with references to external resources. Thus, in order to benefit the most from LLMs’ capabilities, our experiments were directed to document-level relation extraction without requiring us to locate the relations and arguments in the text, or normalizing the entities.

We crafted an instruction prompting the LLM to extract entities and relations according to the EPOP corpus schema (Tables 1 and 2) and to return the output in JSON format.

**Table 1:** Number of entities of each type in the training and development set of the EPOP dataset

Entity type	# Training	# Development
*Disease*	234	148
*Location*	1042	485
*Pest*	908	338
*Plant*	663	347
*Vector*	78	32
**Total**	**2925**	**1350**

**Table 2:** Number of binary relations of each type in the training and development set of the EPOP dataset

Relation type	# Training	# Development
*Causes*	66	35
*Affects*	141	74
Has been found on	441	181
*Located in*	1210	567
*Transmits*	36	13
Total	1894	870

**Fig. 1:**
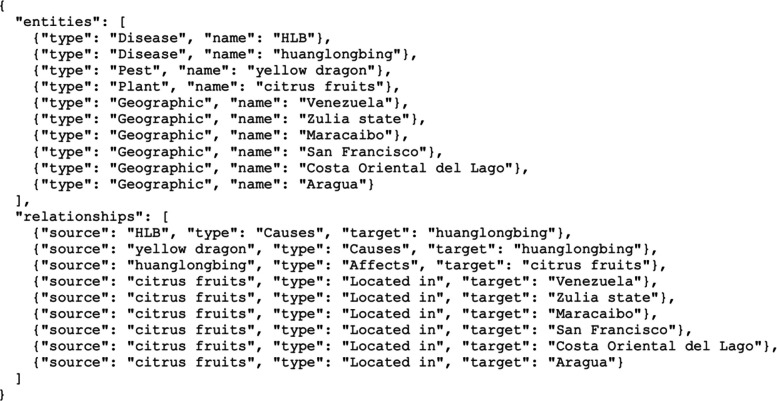
Example of expected output of LLM for document-level relation extraction in the EPOP–DocRE corpus

An example of the expected output is shown in Fig. 1. Relations are expressed as triplets〈source, type, target〉, where *source* and *target* are the surface forms of relation arguments, and *type* is the relation label. Only the relation subpart of the output is evaluated. However, entities are explicitly requested in the instruction, as we found that including this intermediate step leads the LMMs to produce more consistent and accurate relation predictions.

### Reference data

For our experiments on relational information extraction, we selected the EPOP corpus on the epidemiomonitoring of plants (10.57745/ZDNOGF) that is representative of relational IE challenges in real-life applications. The EPOP dataset features a rich annotation schema with seven named entity types and seven binary relation types with strict argument type constraints. To maintain an appropriate level of difficulty for evaluating the link between accuracy and consistency, we focused on five entity types and five relation types. The dense annotation set includes nested and discontinuous entities, synonyms including abbreviations and acronyms, as well as short and long-distance overlapping relationships. Tables 1 and 2 show the number of entities and relations, respectively, in the training and development sets that we used.

Table 3 summarizes the semantics of the relationships and the entity type constraints of their source and target arguments.

**Table 3:** Semantics of binary relation types of the EPOP corpus and their argument constraints

Relation type	Semantics	Source entities	Target entities
*Causes*	A pest that causes a disease	*Pest*	*Disease*
*Affects*	A disease that affects a host plant	*Disease*	*Plant*
*Has been found on*	An organism that was observed on/in another	*Pest* or *Vector*	*Vector* or *Plant*
*Located in*	An organism that was observed at a location	*Pest*, *Plant* or *Vector*	*Location*
*Transmits*	A vector that carries and transmits a pest or a disease	*Vector*	*Pest* or *Disease*

**Fig. 2:**
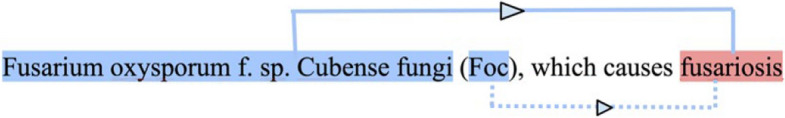
The *Fusarium oxysporum f. sp. Cubense fungi* and *Foc* entities are members of the same coreference set playing the same semantic role as the agent of the *fusariosis* disease

The annotation dataset also includes coreference sets of the text entities that are semantically and rhetorically equivalent. Figure 2 displays an example of a species name and its acronym belonging to a common coreference set.

The coreference information is highly relevant to our objective of distinguishing between recoverable semantic variations in LLM predictions—those that preserve meaning—and disruptive errors. Likewise, the entity-linking annotations in EPOP for four entity types (Pest, Vector, Plant, and Location) from two external resources (NCBI taxonomy and GeoNames) provide information on the semantic similarity between entity occurrences. We used these normalization annotations to identify acceptable semantic or lexical variations. The entity mentions that have the same class identifier are counted as similar. For instance, the species names *Bactrocera dorsalis* and *oriental fruitfly* are respectively the scientific name and the common name of the same species, thus they are annotated with the same identifier of the NCBI taxonomy (27457). It is worth noting that all entities in the same coreference set are of the same type and are normalized with the same identifier.

Coreference sets and normalization information in the EPOP corpus were then used to assess the significance of variations in the LLMs’ outputs for our DocRE task, moving beyond surface-level comparison with the reference annotations. The objective is to mitigate inconsequential variations to prevent them from affecting the comparison between predicted relations and the reference relations.

### Reference data for the DocRE task

To this end, we preprocessed the EPOP annotations to build sets of acceptable entity surface forms for all relation arguments. Since the original corpus is designed for text-bound IE, we further aggregated the annotations to derive the document-level relations suitable for the DocRE task.

More specifically, the EPOP corpus contains text-grounded annotations, thus the same relation may be mentioned multiple times within a single document. These duplicate mentions need to be aggregated into a unique relation per document. The preprocessing of the EPOP annotations is done in three steps for each document to support both entity surface form and relation aggregation:

1. All relations of the document are collected.
2. For the source and target arguments of each relation, the set of acceptable entity forms is collected. Considered acceptable are the surface forms of all entities of the document that either belong to the same coreference set (line 1 in Table 4) or that are normalized with the same class identifier (line 2 in Table 4). The result is a collection of relations with a set of entity forms for each argument. In this step, all forms are case-folded and space-normalized.
3. Equivalent duplicate relations are removed. Two relations are considered duplicates if they share the same type and have identical sets of source and target arguments (line 3 in Table 4).

**Table 4:** EPOP annotation processing for the DocRE task

	EPOP corpus annotation	DocRE version of EPOP
Semantic equivalence set built using coreference sets	(e^1^,r^1^,e^2^) (e^3^,r^1^,e^2^) CorefSet1(e^1^,e^3^)	({e^1^, e^3^}, r^1^, e^2^)
Semantic similarity sets built using entity-linking	(e^1^,r^1^,e^2^) (e^3^,r^1^,e^2^) Class(e^1^,C^1^) Class(e^3^,C^1^)	({e^1^, e^3^}, r^1^, e^2^)
Duplicate relations	(e^4^,r^1^,e^5^) (e^4^,r^1^,e^5^)	(e^4^,r^1^,e^5^)

*r*^*i*^ denotes the relation type, *e*^*i*^ denotes the surface form of relation arguments, *C*^*i*^ denotes the class ofthe entity mention

This adapted version of the EPOP dataset tailored for the DocRE task with a reduced number of entity and relation types (from seven to five) is called EPOP-DocRE from now on. It contains 1652 reference relations, of which 1244 involve semantically similar sets of argument entities. For example, in document 100,011, the sets {*Japanese beetle, Popillia japonica, Japanese beetles*} and{*Freiburg, Freiburg area*} are the source and target arguments of a *Located in* relationship.

### Format error handling

The instruction prompts the LLMs to produce a JSON formatted output. In cases where the produced JSON is malformed, a post-processing step is applied to correct superficial format errors (e.g., extra commas, unmatched delimiters) to enable parsing and evaluation. Outputs that remain invalid after correction are excluded from subsequent processing steps because they typically correspond to easily detectable failures and would introduce noise in the evaluation of the correlation between accuracy and consistency.

### Accuracy measure

Predicted relations are matched to reference relations by comparing the type of the relation and the surface forms of their arguments. A predicted relation is counted as a true positive if it has the same type as a reference relation, and its source and target forms match any of the acceptable forms for the corresponding source and target in the reference. If several predicted relations match the same reference relation, only one is counted as a true positive, and the duplicates are ignored. Predicted relations that do not match any reference are counted as false positives, while reference relations that do not match any predicted relation are counted as false negatives. The counts are then used to compute Recall, Precision, and F_1_ scores for each run on each document. As the experiment is repeated for each model-document pair, we retain the average F_1_ of the well-formed answers as the metric of model accuracy.

### Consistency measure

For each document, the consistency is measured using Fleiss’ Kappa metric on all well-formed predictions [11]. Fleiss’ Kappa is a standard inter-annotator agreement metric that generalizes Cohen’s Kappa for more than two raters. In this study, multiple generations of the same model on the same input are treated as different raters, and each unique relation triplet appearing across runs is treated as a subject for consistency evaluation. To construct the Kappa rating matrix, each relation triplet is assigned a binary label for each generation run (1 if the relation is present in that run, 0 otherwise), yielding an N×2 matrix over all unique predicted relations. Here, N represents the total number of distinct relation triplets, while 2 corresponds to the two possible rating categories (presence vs. absence). Consistency is then quantified using the Fleiss’ Kappa coefficient: $\kappa = \frac{\bar{P} - P_e} {1 - P_e}$, where P denotes the observed agreement among repeated generations and P_e_ represents the expected agreement by chance, estimated from marginal category frequencies. This provides a single interpretable measure of prediction stability across runs. However, the comparison of arguments is strict and does not attempt to retrieve equivalent relations with different surface forms. Indeed, this study attempts to test whether the consistency can be used as a proxy for accuracy, and thus cannot exploit the gold annotation.

### Complexity and document length measure

One of our objectives is to measure to what extent consistency is influenced by the length of the LLM input and the inherent complexity of the task. We selected two metrics to quantify them. Complexity was measured by counting the number of entities and relations in the annotated document, with repetitions included, as they may indicate a more complex semantic structure. Document length was measured by byte count, as it better reflects token definitions in LLMs than word count.

### Correlation measures

We selected the appropriate correlation measures by analysing the accuracy and consistency scores of the four models applied to the EPOP data. As shown in Supplementary Figs. A1 and A2, the distributions of accuracy and consistency scores across the four models were found to closely resemble Gaussian curves. Subsequently, Kolmogorov–Smirnov (K–S) tests were performed on both sets of scores. The results indicated that both distributions passed the K–S test, confirming that they follow a Gaussian distribution (Supplementary Table A1). Therefore, both parametric and non-parametric correlation measures can be applied. Pearson correlation, Spearman rank correlation, and Kendall’s tau test were selected to assess the relationship between consistency and accuracy.

### Experimental setting

We conducted experiments with four widely used LLMs: GPT-4o-mini (https://openai.com/index/gpt-4o-mini-advancing-cost-efficient-intelligence/), DeepSeek-V3 (https://github.com/deepseek-ai/DeepSeek-V3), moonshot-v1-32 k (Kimi) (https://platform.moonshot.cn/), and qwen3-32b with non-thinking mode (Qwen3 [12]). All models were accessed through OpenAI’s Python API.

*Temperature* and *top-p* are critical hyperparameters in our study that influence the diversity and stability of model outputs. To ensure reliable results, we swept different values on a subset of the EPOP dataset to identify settings that maximize correct and valid answers. We analyzed the impact of these parameters on consistency and accuracy as shown in Supplementary Figs. A3 and A4, increasing temperature consistently reduced model consistency and slightly decreased accuracy. Similarly, top-p values above 0.5 led to further drops in both metrics. Based on these findings, we fixed the temperature at 0.2 and top-p at 0.1 in all experiments.

Meanwhile, we examined the impact of the number of sampling repetitions on accuracy and consistency evaluation. Repeated sampling involves generating multiple outputs from the same prompt to assess the stability of the model’s predictions. While repeated trials slightly improved consistency, they had minimal effect on accuracy, as shown in Supplementary Fig. A5. To trade off the evaluation reliability and computational cost, we fixed the number of repetitions to 5 in all experiments, which also aligns with prior studies [7].

For prompt design, we compared two settings: one where the LLM was instructed to perform only the relation extraction task (RE-only), and another where it was asked to perform both named entity recognition and relation extraction (NER&RE). Indeed, NER is often a prerequisite for RE, and [Li] shows LLMs perform better when prompted for both named entity recognition and relation extraction vs. only relation extraction. As shown in Supplementary Fig. A6, the RE-only setting led to a significant drop in both accuracy and consistency in most cases, which confirms previous observations. Consequently, all models were prompted to perform both tasks to ensure better overall performance. The exact prompts used in these experiments are provided in Supplementary Figs. A7 and A8.

## Results

The average accuracy and consistency scores obtained by the four models on the EPOP-DocRE dataset are given in Table 5.

**Table 5:** Macro-average accuracy and consistency scores of models

Model	Average Accuracy	Average Consistency
GPT-4o-mini	**0.5274**	**0.5169**
Kimi	0.5509	0.5108
DeepSeek-V3	0.6056	0.4790
Qwen3	0.5236	0.2627

Mean F1 scores (Accuracy) and Kappa scores (Consistency) for four models

Among the evaluated models, GPT-4o achieved the highest average accuracy and consistency scores overall. Pearson correlation, Spearman’s rank correlation, and Kendall’s tau tests were used to assess the correlation between accuracy and consistency across the four models. Since an accuracy score and a consistency score were computed for each LLM on each document, the correlation analysis was performed at the document level, treating each document as an individual observation. As presented in Table 6, all four models demonstrate weak correlations between 0.10 and 0.30 in all tests, indicating a limited association between accuracy and consistency. DeepSeek-V3 shows the highest correlation, with a Spearman coefficient of 0.3017. For all models, the p-values are statistically significant.

**Table 6:** Statistical correlation between accuracy and consistency

		GPT-4o-mini	DeepSeek-V3	Kimi	Qwen3
Pearson test	Correlation coefficient	0.2230	0.2552	0.2849	0.2980
	*p*-value	3.99e−3	9.38e−4	2.08e−4	1.01e−4
Spearman’s rank test	correlation coefficient	0.2019	0.2587	0.2795	0.2049
	*p*-value	9.29e−3	7.92e−4	2.76e−4	8.29e−3
Kendall’s tau test	correlation coefficient	0.1420	0.1884	0.1978	0.1453
	*p*-value	8.67e−3	5.02e−4	2.58e−4	6.03e−3

Results of Pearson correlation, Spearman’s rank correlation, and Kendall’s Tau tests evaluating the correlation between accuracy and consistency of outputs across four models on the EPOP-DocRE dataset

Table 7 reports the average accuracy and consistency scores for the four models, where accuracy is computed without using semantic equivalence among argument entities derived from coreference and entity-linking information. As expected, accuracy dropped significantly for all models ranging from 23 points for Qwen3 to 30 points for DeepSeek.

**Table 7:** Macro-average accuracy without semantic equivalence mapping

Model	Average Accuracy
GPT-4o-mini	0.2686
Kimi	0.2974
DeepSeek-V3	**0.3104**
Qwen3	0.2919

However, the strict string-matching evaluation of accuracy has limited impact on the correlation between accuracy and consistency, which remains weak between 0.10 and 0.30 across all tests, as shown in Table 8. Most values show a slight decrease (highlighted in blue), with only two showing a minor increase (in red).

**Table 8:** Statistical correlation between accuracy and consistency without semantic equivalence argument merging

		GPT-4o-mini	DeepSeek-V3	Kimi	Qwen3
Pearson test	correlation coefficient	**0.1763**	**0.1663**	*0.2994*	**0.1707**
	*p*-value	2.35e−2	3.27e−2	9.33e−5	2.84e−2
Spearman's rank test	correlation coefficient	**0.1803**	**0.1617**	**0.2747**	**0.0974**
	*p*-value	2.05e−2	3.56e−4	3.79e−2	0.213
Kendall's tau test	correlation coefficient	**0.1337**	*0.2025*	**0.1284**	**0.0673**
	*p*-value	1.71e−2	2.75e−4	1.87e−2	0.2137

Boldface coefficients represent lower correlation values compared to results with semantic equivalence merging, whereas italicized coefficients indicate higher values

### Impact of document complexity and length

**Fig. 3:**
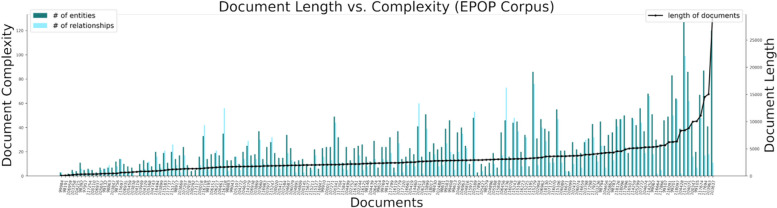
Document length and complexity in the EPOP Corpus. Document length is measured in bytes; complexity is defined as the total count of entities and relations per document, including duplicates. Documents are ordered by increasing length

**Fig. 4:**
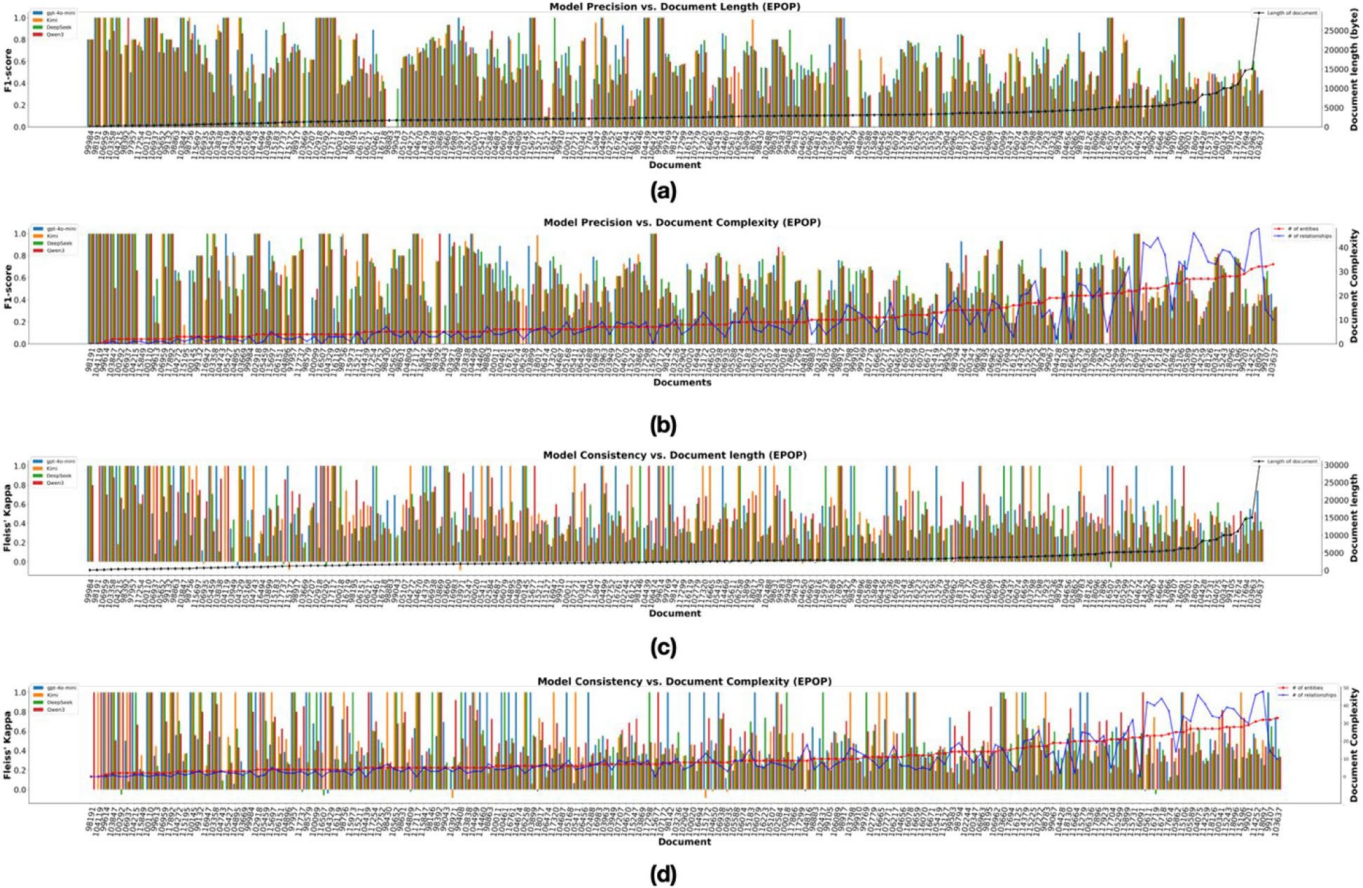
Model precision and consistency across document length and complexity in the EPOP corpus. Subfigures **a** and **b** show how model prediction accuracy varies with document length and complexity, respectively. Subfigures **c** and **d** depict the relationship between prediction consistency and document length and document complexity, respectively. Specifically, documents in **a** and **c** are sorted by document length, while **b** and **d** are sorted by document number of entities (red line), while the number of relationships is shown by the blue line

We also examined the relationship between document complexity and length, as well as their effect on model output consistency and accuracy. As expected, document complexity—measured by the number of entities and relations to be predicted—increases with document length (Fig. 3). In most cases, both accuracy and consistency of the LLMs showed a general decline as document length and complexity increased (Fig. 4a–d*).

**Fig. 5:**
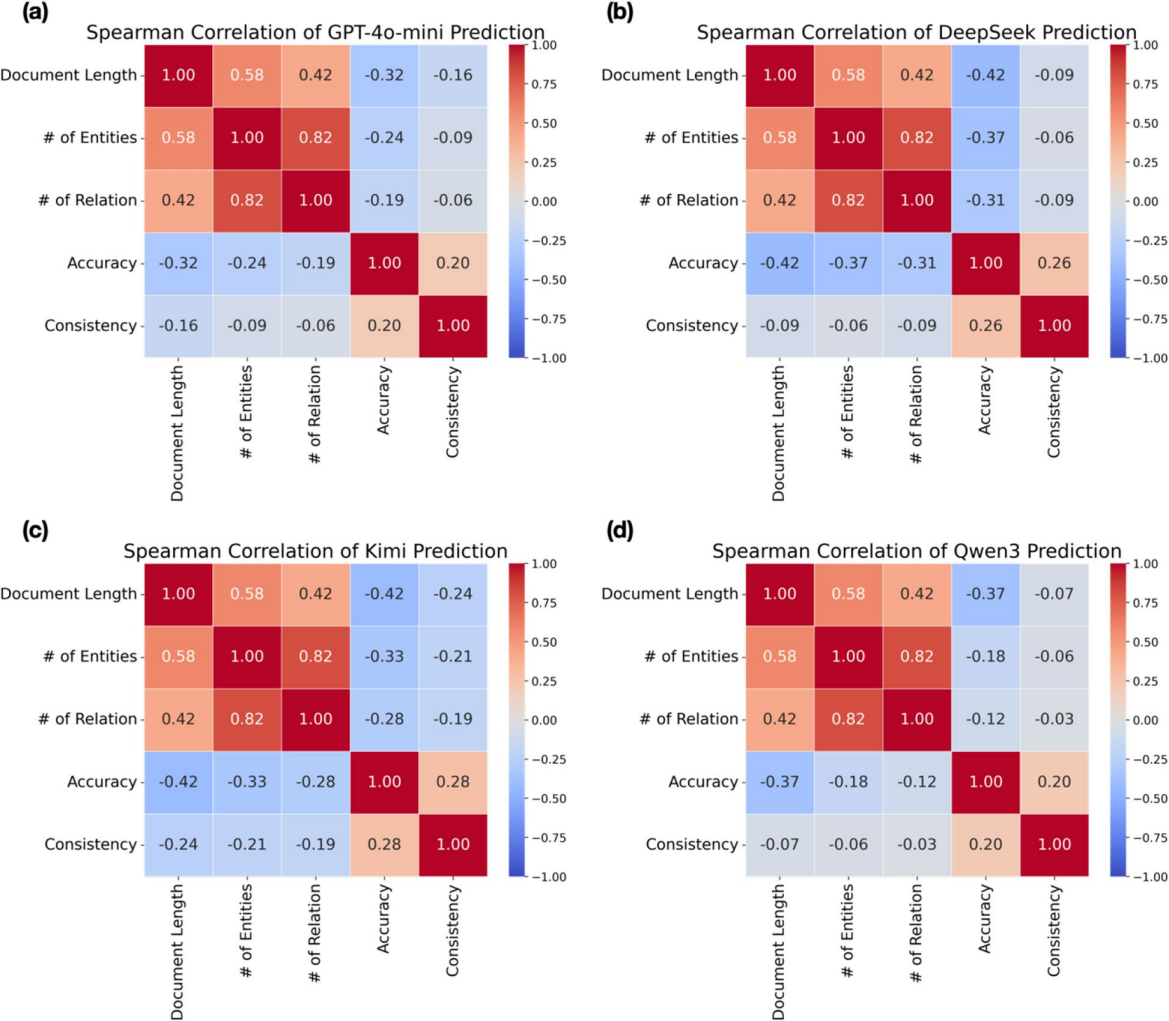
Spearman correlation between precision, consistency, and document attributes. Spearman rank correlation coefficients are shown for document length, document complexity, prediction accuracy, and consistency across four models

To validate our visual observations, we applied Spearman’s rank tests to assess correlations among document length and complexity variables, as well as their relationships with model accuracy and consistency across the four models (Figs. 5, A9, and A10). As expected, the test revealed a strong positive correlation between document length and complexity. Both document variables were negatively correlated with accuracy and consistency scores. Among the complexity components, the number of entities showed an even stronger negative correlation with model performance than the number of relationships.

### Well-formed outputs

Before evaluation, the model output was processed to correct superficial format errors. The second column of Table 9 gives the proportions of recoverable format errors per model, computed over the 825 answers (165 documents, 5 runs per document). The Kimi model was the one that adhered to the format most accurately.

**Table 9:** Proportion of format errors in model outputs

Model	% recoverable formaterrors	% unrecoverable format errors	% Valid outputs
GPT-4o	0,059	0,000	1.000
DeepSeek	0,040	0,000	1.000
Kimi	0,019	0,001	0.999
Qwen3	0,976	0,015	0.985

The number of outputs that remained unparseable after applying superficial formatting error correction was low: zero for GPT and DeepSeek, one for Kimi, and twelve for Qwen. Column 3 of Table 5 displays the proportion of the unrecoverable formatting errors. In our experiments, there was no case where all five runs of Qwen produced output with an invalid format. Typical malformed outputs include missing property names in JSON objects representing relation instances (Qwen3) and excessively long, repetitive segments that terminate prematurely (Kimi). Qwen3 produced the highest number of both recoverable and unrecoverable formatting errors.

## Discussion

Our study provides new insights into the relationship between consistency and accuracy in relational information extraction (RE) using generative large language models (LLMs). While consistency has been proposed as a signal of reliability in generative settings, our findings indicate that high consistency does not strongly correlate with high accuracy. Across all tested models, the correlation between consistency and accuracy was weak, suggesting that consistency alone should not be relied upon as a proxy for prediction quality in relational IE tasks.

The document-level relation extraction (DocRE) task emerges as a more appropriate benchmark for generative LLMs than traditional text-bound IE. By abstracting relations to a document level, DocRE avoids explicit positional alignment and repetition modeling the LLMs’ struggle with in our experiments.

Another important outcome of our analysis is the need to distinguish between shallow, recoverable output variations and deeper, semantic inconsistencies. Minor typographical and formatting errors should not be conflated with semantically incorrect predictions. Similarly, variations that preserve meaning—such as those arising from synonymy or coreference—should not penalize models during evaluation. The EPOP corpus, which includes both normalization and coreference annotations, allowed us to account for many of these recoverable variations in our accuracy and consistency assessments. Ignoring such equivalence leads to a substantial drop in accuracy scores across all models. This outcome appeals for the use of external lexical and semantic resources to make the most of LLMs for the purpose of knowledge base construction in specific domains.

Looking forward, we advance that evaluation metrics for generative IE should evolve to better reflect semantic equivalence. While coreference and synonymy offer a useful starting point, there is considerable potential to develop automatic semantic matching techniques that go beyond these surface-level signals. Integrating such capabilities into future evaluation frameworks will improve both model training and assessment by rewarding semantically correct outputs—even when their surface forms vary.

However, the correlation between accuracy and consistency remains weak regardless of whether semantic equivalence is considered. This suggests that consistency alone is not a reliable proxy for accuracy in generative information extraction tasks, even when semantic nuances are taken into account.

### Future work

This study focused on *hard prompting* settings, where generative large language models (LLMs) are used without task-specific fine-tuning. While this approach allows for broad applicability, it may not reflect the performance characteristics of *fine-tuned LLMs*, which could exhibit different consistency–accuracy dynamics. Fine-tuning might improve task-specific behavior, enforce stronger adherence to structural constraints, and reduce output variability—potentially leading to different conclusions regarding the relationship between consistency and accuracy.

Our evaluation was limited to a single domain—plant health—within a document-level relational information extraction (DocRE) task. Although this domain is representative of real-world relation extraction challenges (e.g., long-range dependencies, entity variability), the extent to which our findings generalize to other domains (e.g., biomedical, legal, or finance) or to other structured tasks (e.g., event extraction, question answering) remains to be explored. Future work should assess whether the observed weak correlation between consistency and accuracy holds across different data characteristics, annotation schemes, and task requirements.

As previously observed by other work [13] we observed in our experiments that LLMs frequently violate argument-type constraints, especially when the relationship or entity types are semantically or rhetorically close, such as Disease and Pest types in the Transmits relation, where the disease is used in place of the pathogen (e.g., “*Cassava brown streak virus* disease transmitted by whiteflies” instead of “*Cassava brown streak virus* transmitted by whiteflies”). Our task used a constrained schema where such confusions are counted as errors. The consistency–accuracy correlations we report may not hold in settings with looser schemas or with post-processing adjustments of the LLM answers [10]. This highlights the need for further investigation into the effect of schema design on LLM stability and accuracy.

In this study, consistency was measured by comparing raw outputs across multiple runs, using surface-level string matching. While simple, this approach overlooks cases where outputs differ only superficially yet remain semantically consistent. More advanced consistency metrics could address this limitation by identifying meaningful consistency through semantic similarity. This could be done by incorporating semantic similarity through coreference resolution, synonym detection, structured relation alignment, or referencing external knowledge bases as done for accuracy evaluation. Such advancements would improve their ability to predict model reliability in downstream knowledge-intensive applications. However, implementing these approaches during inference would entail substantial additional effort.

## Supplementary Information

Supplementary Material 1.

Supplementary Material 2.

## Data Availability

Prompt: https://github.com/YaoXinZhi/BLAH9-Consistency-Accuracy-LLM-EPOP/tree/main/prompt//. Corpus: https://github.com/YaoXinZhi/BLAH9-Consistency-Accuracy-LLM-EPOP/tree/main/dataset//. Prediction results: https://github.com/YaoXinZhi/BLAH9-Consistency-Accuracy-LLM-EPOP/tree/main/prediction//. Code: https://github.com/YaoXinZhi/BLAH9-Consistency-Accuracy-LLM-EPOP/tree/main/script.
