# Supplementary material for: Consistency–accuracy correlation in hard-prompted LLMs for entity and relation extraction: empirical findings from plant-health data
Source: Genomics Inform. 2026 Feb 10;24:3. doi: 10.1186/s44342-025-00063-2 (PMC12888769; doi:10.1186/s44342-025-00063-2)
Supplement: Supplementary file 1 — Supplementary Material 1. [file 44342_2025_63_MOESM1_ESM.pdf]

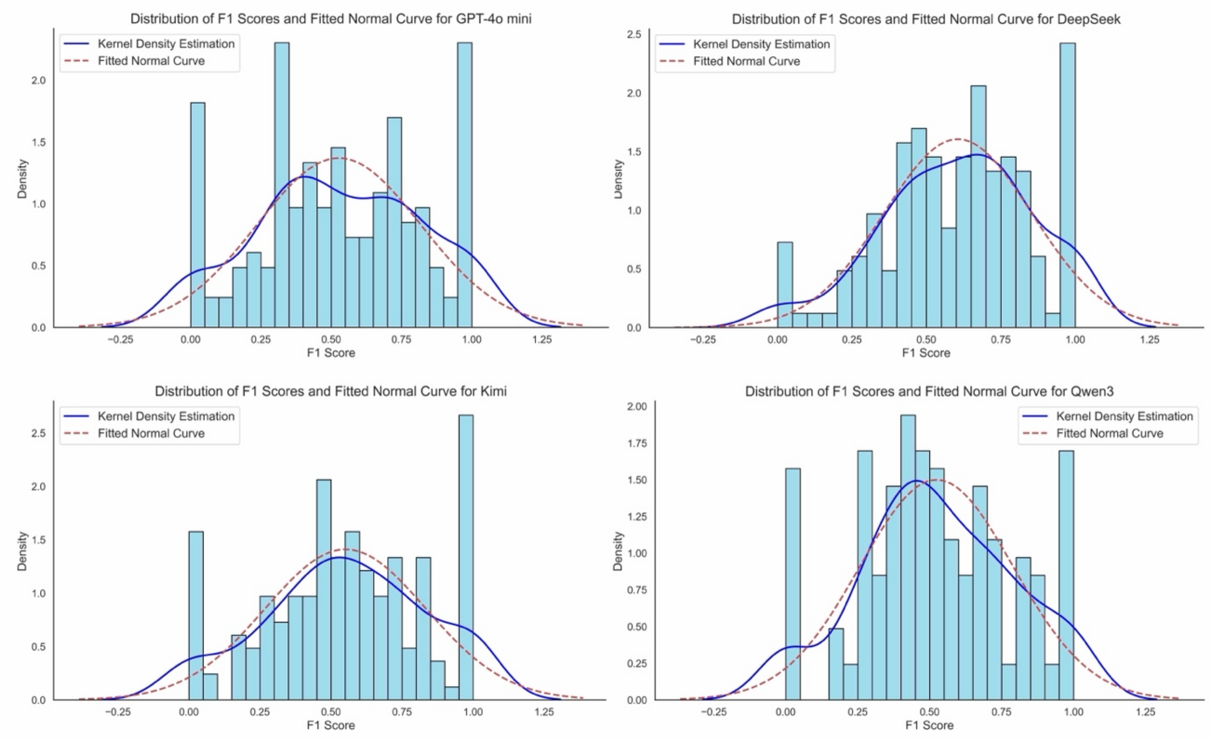

Supplementary **Fig. A1**

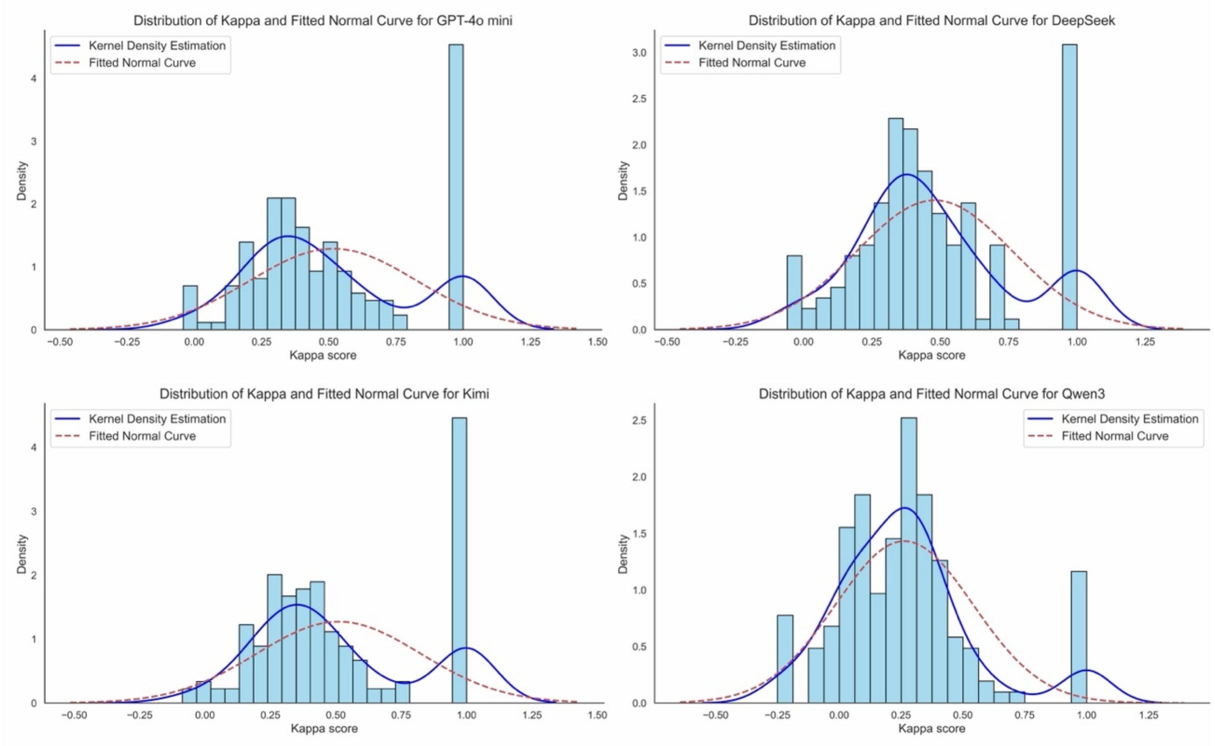

Supplementary **Fig. A2**

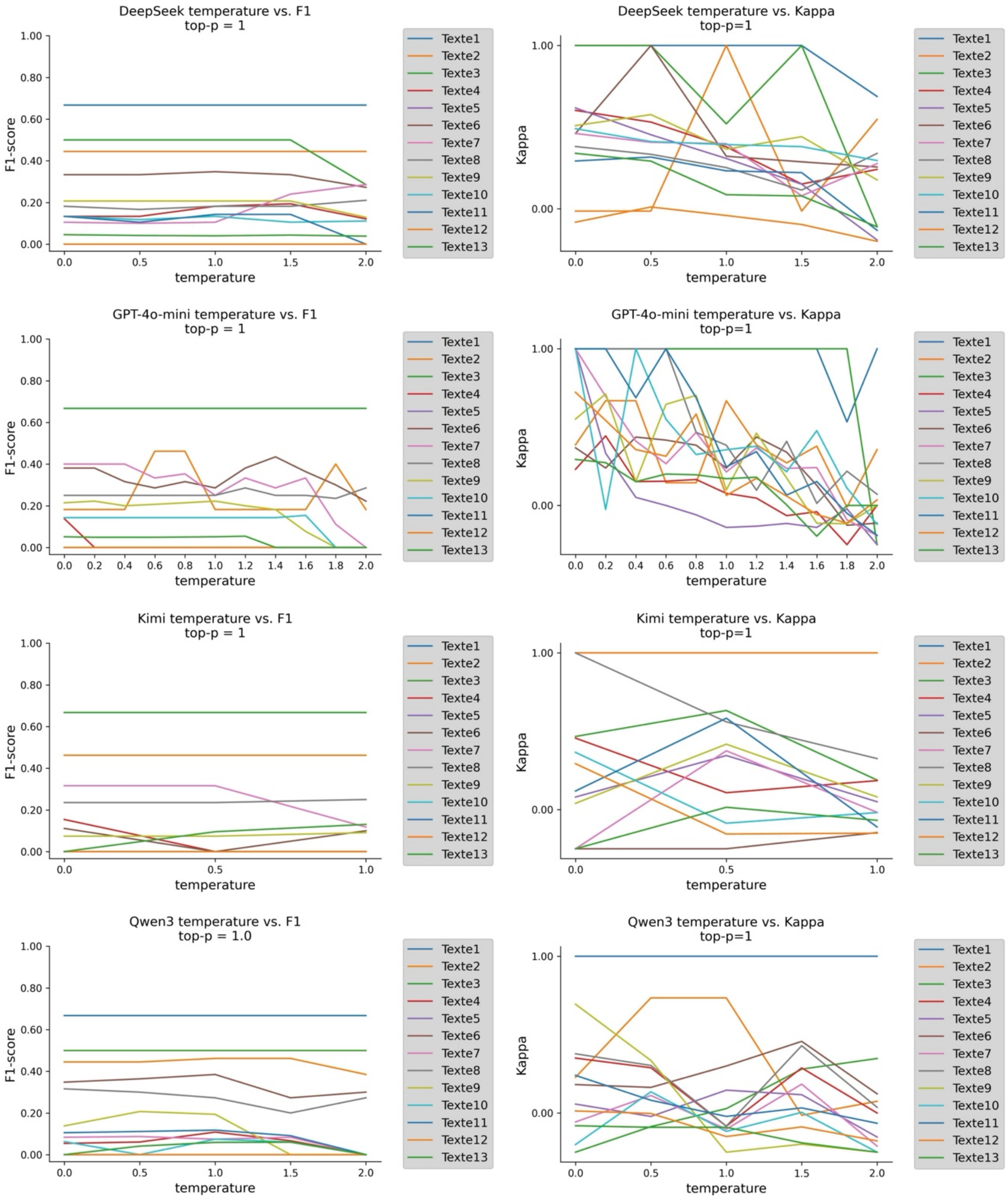

Supplementary **Fig. A3**

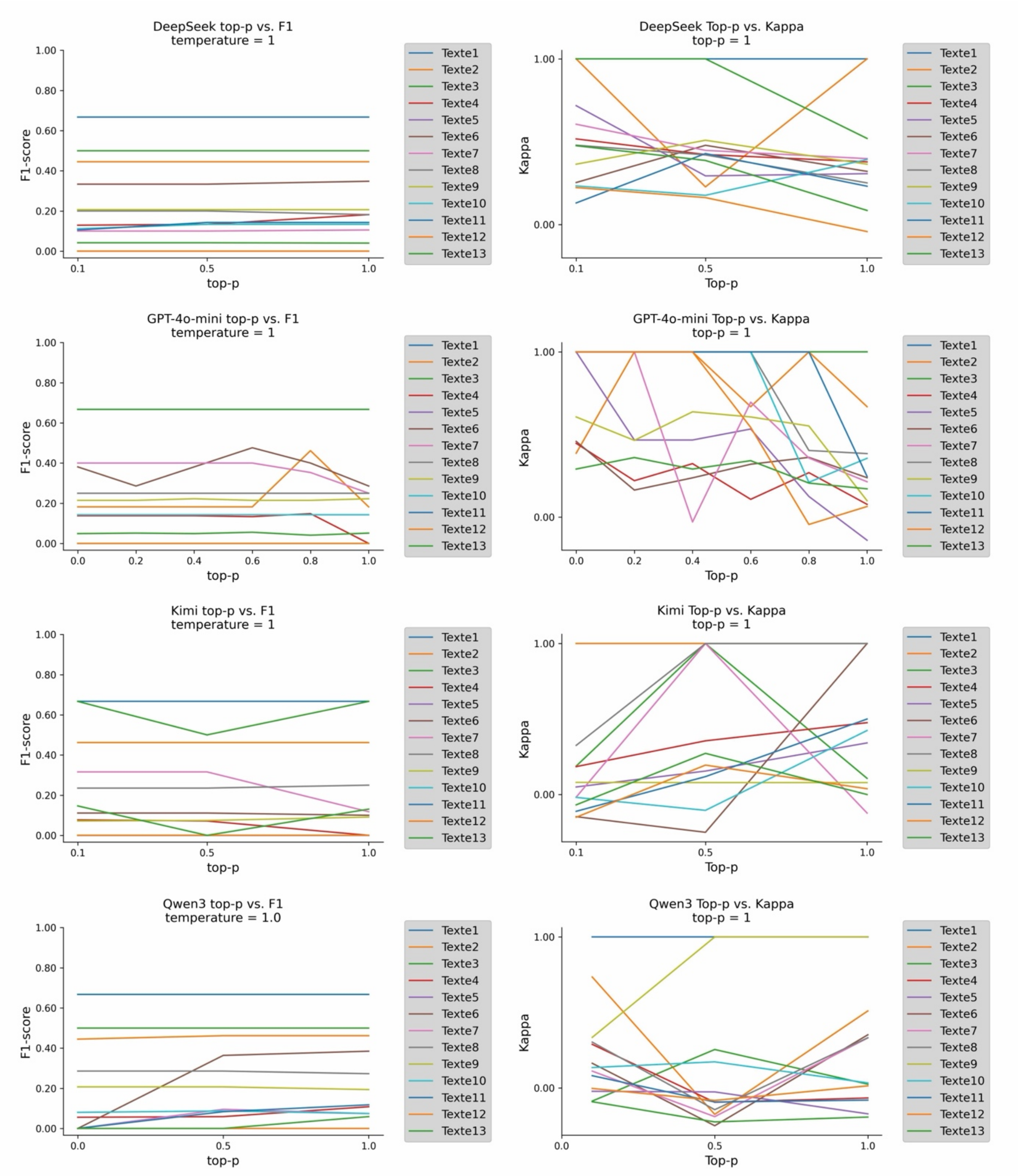

Supplementary **Fig. A4**

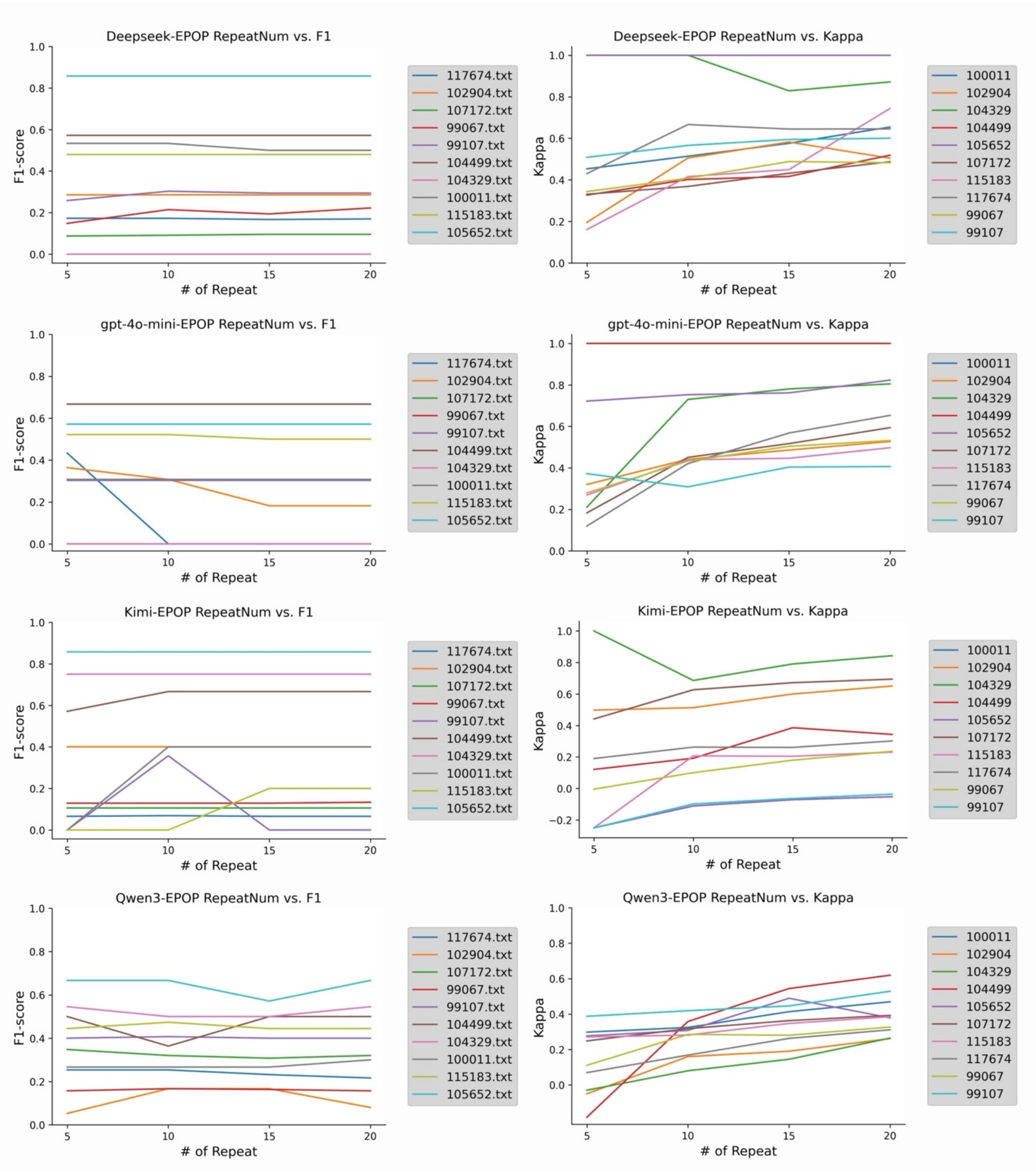

Supplementary **Fig. A5**

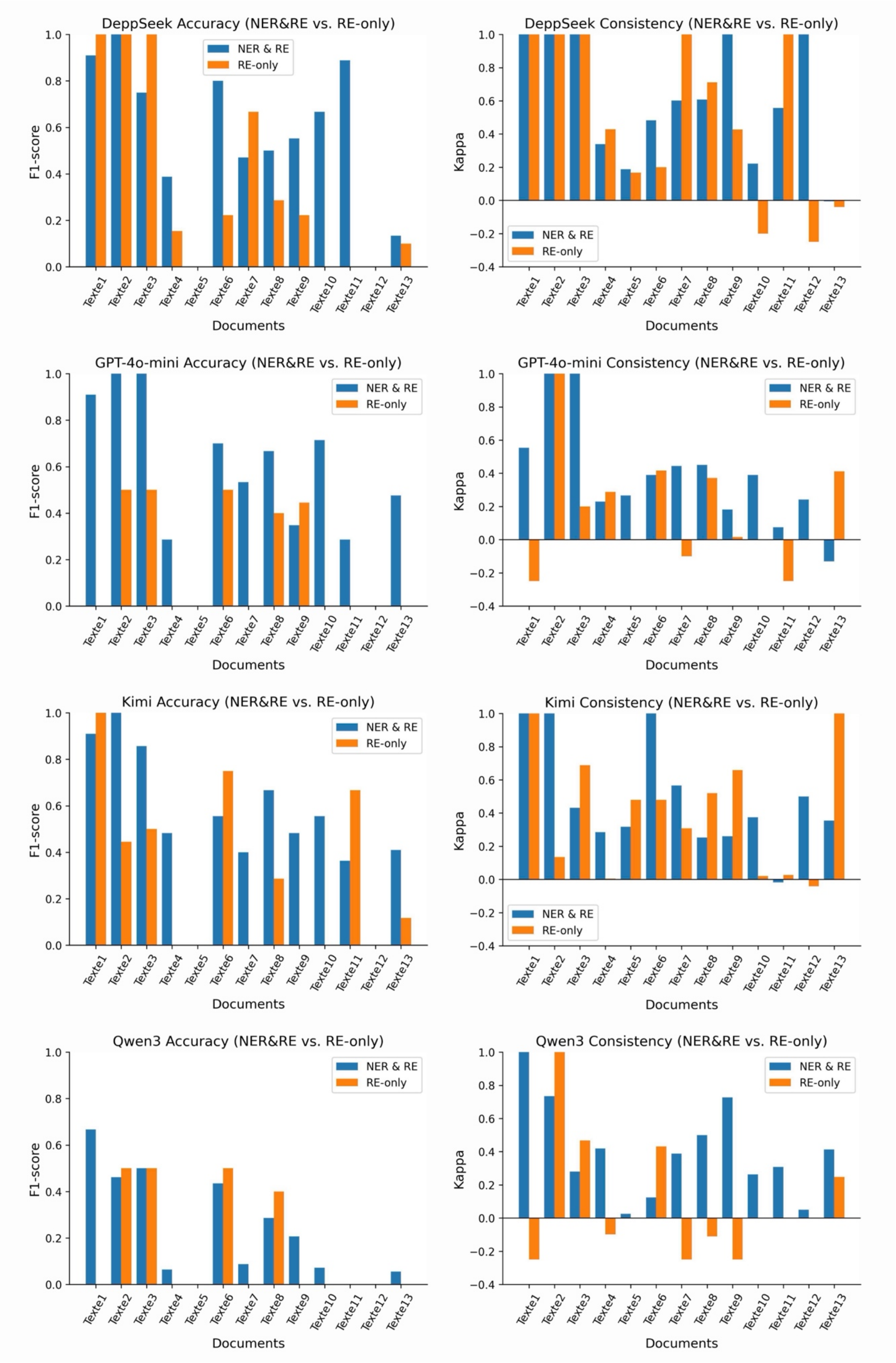

Supplementary **Fig. A6**

A. Task RE only

You are an information extraction system. Your task is to identify relationships between entities in a given text.

Information to be extracted

Relationships

- Located in: Describes the geographical location where a plant, a pest, or a disease is found.
  Example: "The pest 'Bursaphelenchus xylophilus' is located in Japan."
- Causes: Indicates that a pest is responsible for causing a particular disease.
  Example: "The pest 'Bursaphelenchus xylophilus' causes pine wilt disease."
- Have been found on: Describes instances where a pest, or a vector has been detected on a specific plant.
  Example: "The pest 'Bursaphelenchus xylophilus' has been found on pine trees."
- Affects: Indicates that a disease affects a plant.
  Example: "Pine wilt disease affects pine trees."
- Transmit: Describes instances where a specific vector transmits a pest.
  Example: "The vector 'Monochamus alternatus' transmits the pest 'Bursaphelenchus xylophilus'."

Relationship arguments

The types of entities that are arguments of the relationships are fixed. The argument type for each relationship is specified below in brackets. The type of the first argument is before the relationship. The type of the second argument is after the relationship. I have denoted alternative entity types by a comma.

- [plant, pest, disease] Located in [geographic]
- [pest] Causes [disease]
- [pest, vector] Have been found on [host plant].
- [Disease] Affects [Plant]
- [Vector] Transmit [Pest]

Avoiding redundancy

Remove redundant relationships where the arguments involve entities that play the same role in the text.

Format

Each relationship appears as an entry in a JSON-formatted list. Each relationship must have three properties, source argument, relationship label and target argument.
The JSON-formatted list can remain empty.

Additional instructions, special cases

Only include entity names that appear in the text. Do not modify their writing.
The entity names must be precise, and avoid generic terms.
Entity names that denote living organisms i.e. plants, pest and vector, must be extracted if they denote genera, species or subspecies. This includes genetically modified organisms.
Extract all entities of the requested types, including those that are not arguments of any relationship.
Ensure the relationship arguments are entities that you have extracted.

Supplementary **Fig. A7**

Task NER & RE

Your task is to identify all named entities and relationships from text, adhering strictly to the schema below.

Information to be extracted

Entities

Entities must be of the following types :

- Pest: a specific plant pest that is susceptible to infect or cause damage to a host plant
- Vector: an insect that is susceptible to transmitting a pest to a host plant
- Plant: a plant
- Disease: a plant disease
- Geographic: political or physical location

Relationships

- Located in: Describes the geographical location where a plant, a pest, or a disease is found.
  Example: "The pest 'Bursaphelenchus xylophilus' is located in Japan."
- Causes: Indicates that a pest is responsible for causing a particular disease.
  Example: "The pest 'Bursaphelenchus xylophilus' causes pine wilt disease."
- Have been found on: Describes instances where a pest, or a vector, has been detected on a specific plant.
  Example: "The pest 'Bursaphelenchus xylophilus' has been found on pine trees."
- Affects: Indicates that a disease affects a plant.
  Example: "Pine wilt disease affects pine trees."
- Transmits: Describes instances where a specific vector transmits a pest.
  Example: "The vector 'Monochamus alternatus' transmits the pest 'Bursaphelenchus xylophilus'."

Relationship arguments

The types of entities that are arguments of the relationships are fixed. The argument type for each relationship is specified below in brackets. The type of the first argument is before the relationship. The type of the second argument is after the relationship. I have denoted alternative entity types by a comma.

- [plant, pest, disease] Located in [geographic]
- [pest] Causes [disease]
- [pest, vector] Has been found on [plant].
- [disease] Affects [plant]
- [vector] Transmits [pest]

Format

Each named entity and relationship appears as an entry in a JSON-formatted list. The structure is as follows:
Each entity must have two properties, type and name.
Each relationship must have three properties: source argument, relationship label, and target argument.
Any of these JSON-formatted lists can remain empty.

Supplementary **Fig. A8**

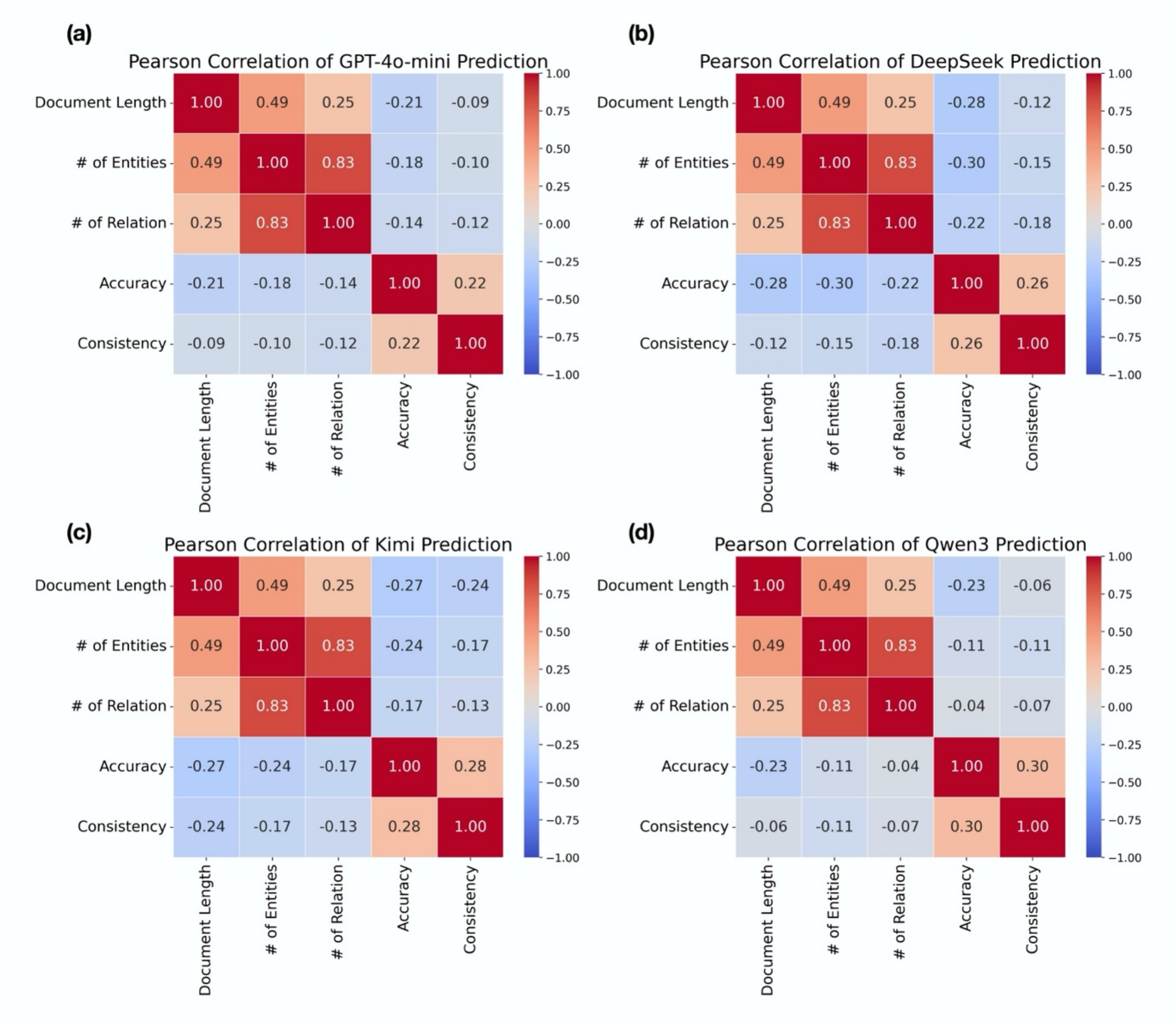

Supplementary **Fig. A9**

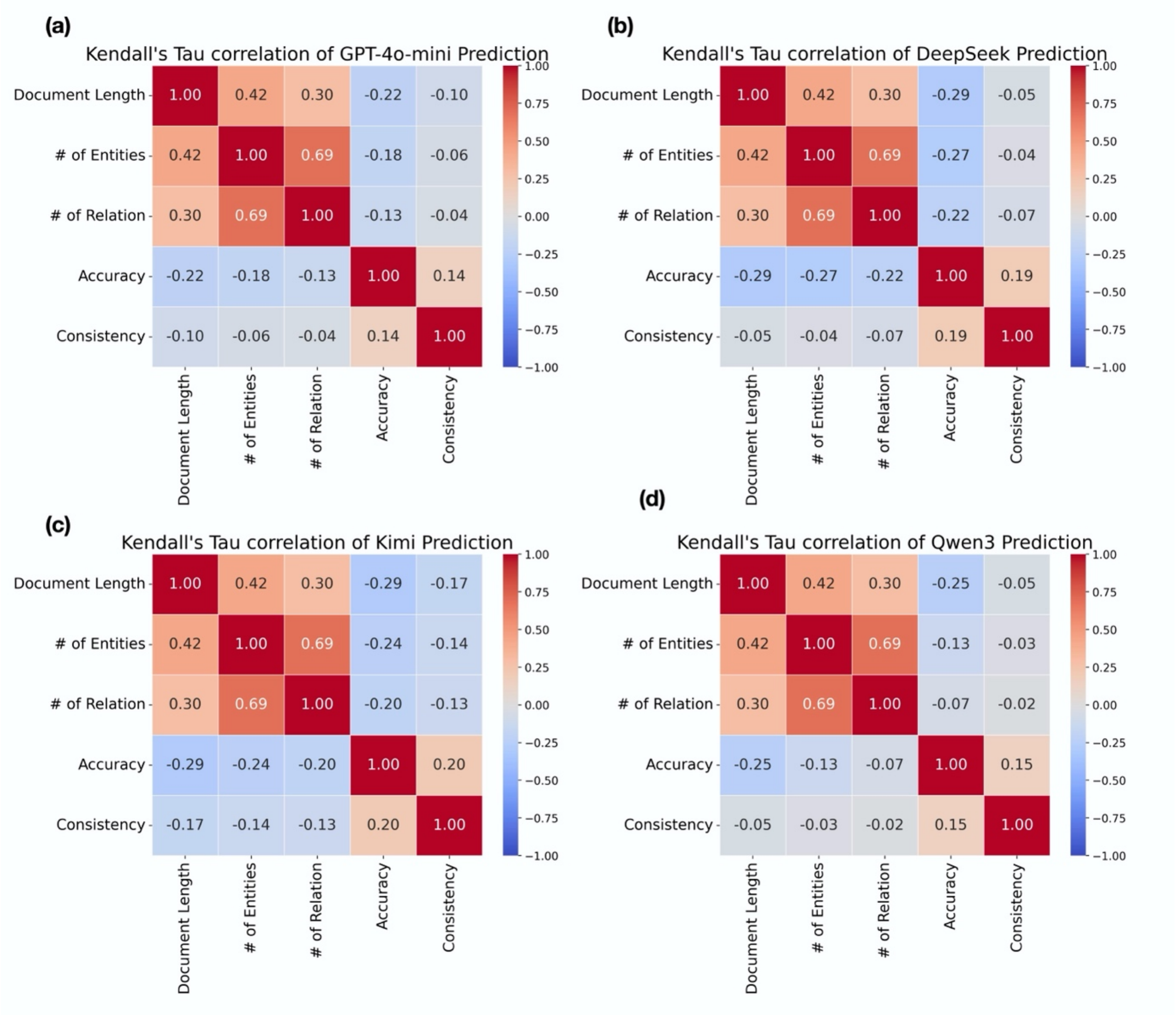

Supplementary **Fig. A10**
