# Supplementary material for: Consistency–accuracy correlation in hard-prompted LLMs for entity and relation extraction: empirical findings from plant-health data
Source: Genomics Inform. 2026 Feb 10;24:3. doi: 10.1186/s44342-025-00063-2 (PMC12888769; doi:10.1186/s44342-025-00063-2)
Supplement: Supplementary file 2 — Supplementary Material 2. [file 44342_2025_63_MOESM2_ESM.docx]

|  | | GPT-4o-mini | DeepSeek-V3 | Kimi | Qwen3 |
| --- | --- | --- | --- | --- | --- |
| Kolmogorov-Smirnov Test for Accuracy | Statistic | 0.1903 | 0.1749 | 0.1599 | 0.1574 |
|  | *p*-value | 1.04e-5 | 6.96e-5 | 3.76e-4 | 4.90e-4 |
| Kolmogorov-Smirnov Test for Consistency | Statistic | 0.1771 | 0.1298 | 0.1829 | 0.1323 |
|  | *p*-value | 5.33e-5 | 6.95e-3 | 2.65e-5 | 5.59e-3 |
